# Illness recognition and care seeking for maternal complications of pregnancy and birth in rural Amhara and Oromia Regional States of Ethiopia

**DOI:** 10.1186/s12884-017-1572-5

**Published:** 2017-11-16

**Authors:** Lynn Sibley, Yared Amare

**Affiliations:** 1 Nell Hodgson Woodruff School of Nursing and Rollins School of Public Health, Emory University, 1520 Clifton Road NE, Room 268, 30322 Atlanta, Georgia; 2 Consultancy for Social Development, P.O. Box – 70196, Addis Ababa, Ethiopia

**Keywords:** Maternal complications, Illness recognition, Care-seeking behavior

## Abstract

**Background:**

Ethiopia has made steady progress in improving maternal health over the decade, yet mortality remains high. The Maternal and Newborn Health in Ethiopia Partnership (MaNHEP) was a 3.5-year project aimed at developing a community-oriented model to improve maternal and newborn survival in rural Ethiopia. Two years after the project ended, we carried out a case study to explore illness recognition and care seeking for complications of pregnancy and childbirth in the project area. This paper describes the results of one component: illness narratives.

**Methods:**

Sampling involved random selection of 12 health facilities from 6 MaNHEP project districts in Amhara and >Oromia regions, and purposive selection of cases from the facility catchment areas. The purposive sample included 17 cases of perceived excessive bleeding, 5 cases of maternal death from any cause, and witnesses to the illness events. Two-person teams facilitated the narrative interviews. Analysis included thematic content analysis of symptoms, causes, decision makers and decision-making, factors facilitating and impeding care seeking, and delineation of care-seeking steps.

**Results:**

Most surviving mothers (and witnesses) perceived the symptoms and seriousness of excessive bleeding; a majority (53%) sought timely biomedical care. Three of five families of mothers who died from causes unrelated to bleeding failed to initially perceive symptoms as serious, yet *all* sought timely appropriate care once they did so. Many of these families took multiple steps to obtain care, leading to delays.. Health worker counseling and proximity to health services facilitated, while certain cultural norms, economic, geographic, and environmental constraints impeded care seeking. Surprisingly, poor quality of care at health facilities was not a barrier.

**Conclusion:**

Mothers and family caregivers are able to recognize and seek timely biomedical care for abnormal bleeding, and for less obvious symptoms of illness. These achievements can be reinforced through continued and focused health education and counseling, reduction of known barriers to care seeking, and improvements in the capacity of the health system to respond to maternal complications with high quality basic and comprehensive emergency obstetric care.

## Background

Globally, the maternal mortality ratio (MMR) declined from 385 deaths per 100,000 live births in 1990 to 216 in 2015, corresponding to an average annual decline of 2.7%. By comparison, the rate of decline for sub-Saharan Africa has been slower, i.e., 2.3% [1, 2]. In Ethiopia, although there has been a substantial reduction in the maternal mortality ratio over the past decade as a result of concerted Government efforts, it remains unacceptably high—353 deaths per 100,000 live births in 2015 [3]. A combination of factors contributed to the high levels of mortality including limited use of maternal and newborn health services. The Ethiopia 2016 Demographic and Health Survey Key Indicator Report shows that, in the 5 years preceding the survey, coverage was improving but still low for focused antenatal, delivery and postnatal care within 2 days of birth (32, 26, and 17%, respectively) [4].

The Maternal Health in Ethiopia Partnership (MaNHEP) was a 3.5-year learning project funded by the Bill & Melinda Gates Foundation to develop a community-oriented model of maternal and newborn survival in rural Ethiopia and to position it for scale up [5]. Operating under the leadership of the Ethiopian Federal Ministry of Health (FMoH) and Regional Health Bureaus (RHB), MaNHEP complemented and strengthened the Government’s flagship Health Extension Program by ensuring delivery of a core package of maternal and newborn health care to achieve United Nations Millennium Development Goals 4 and 5 to reduce child and maternal mortality by two-thirds and three-quarters, respectively, by 2015. The MaNHEP project was implemented by Emory University, in collaboration with John Snow Research and Training Inc., University Research Co. LLC, and Addis Ababa University.

Between 2010 and 2013, MaNHEP staff worked closely with the regional and local Health Extension Program staff, implementing strategies to improve access to and use of maternal and newborn health services. Intervention strategies included refresher clinical training of Health extension workers in safe, clean delivery, community-based maternal and newborn health education for women in the second and third trimester of pregnancy and their family members who would be present at birth; collaborative quality improvement to link communities and health facilities and improve access to care,; and broader behavior change communications to shift norms toward the value of maternal and newborn care and the use of health facilities, and trained providers for maternity care [5]. The MaNHEP 2012 end line survey findings indicate that the project was associated with significant improvements over the 2010 baseline survey findings. The improvements included an increase in completeness of maternal and newborn health care provided by frontline health workers comprised of health extension workers, health development army members, traditional birth attendants (from an average of 52% to 90% of 17 care elements), an increase in frontline health workers’ demonstrate*d* ability to provide the care elements (from an average of 20 to 75% of care elements), and an increase in their confidence to provide care. The project was also associated with an increase in the completeness of maternal and newborn care women reported that they received from frontline health workers (from an average of 32 to 83% of the 17 care elements), and in their trust in frontline health workers to provide care. Women’s receipt of focused antenatal care, delivery care and postnatal care from a skilled provider or health extension worker also increased (from 48 to 86, 9 to 15%, and 29 to 41%, respectively) [5]. At end line, 45% of women who perceived a major maternal complication sought care at a health facility [6]. More than 88% of the project’s 51 *kebeles* had identified and tested local solutions to improve antenatal care registration, birth notification, postnatal care within 48 h of birth. Lastly, there was a significant increase in the average number of days between perinatal deaths [5].

Since 2013, the Ethiopian Federal Ministry of Health has adopted additional strategies to improve access to better quality maternity services including performance review, maternal death review and response, ambulance services at district hospitals and at health centers, increasing midwifery staff, offering a monthly pregnant women’s health education conference and promoting user friendly maternity services. Moreover, the Health Extension Program and the health development army of community health volunteers have become more fully operational [6]. Overall, great effort has gone into improving illness recognition of appropriate care seeking to reduce avoidable maternal death and disability.

In 2014, 2 years after the close of the MaNHEP project, we conducted a mixed- methods nested case study focusing on the current context and status of illness recognition of and appropriate care seeking for both maternal and also newborn complications in the original project area. Funded by the United States Agency for International Development (USAID) Translating Research into Action (TRAction) Project and implemented by University Research Co., L.L.C. (URC), the case study was one of six country case studies that included Ethiopia, India, Indonesia, Nigeria, Tanzania and Uganda. Across the case study sites the teams used a common research protocol that was framed by the Delay Model [7]. The Model posits that delays in appropriate care seeking and care are a result of delays in illness recognition and care seeking decision-making, reaching care, and receiving care on arrival at a health facility. These are, in turn, influenced by socio-cultural, geographic, economic and other logistical constraints to care seeking, and perceived and actual quality of care at health facilities.

In this paper, we detail the main findings from the component of the case study focusing on illness recognition and care seeking for maternal complications and their implications for maternal health policy and programming in Ethiopia.

## Method

### Study site

The study was undertaken in the three districts of Amhara Regional State and three districts of Oromia Regional State that comprised the original MaNHEP project area. The MaNHEP project area included a section of each district served by two health centers and five to six health posts under these health centers. The health centers are staffed by nurses and midwives while the health posts are staffed by two female Health Extension Workers who are responsible for health promotion but also provide some basic curative services. The MaNHEP project area *kebeles* (communities) had a population about 350,000 [5]. For the purposes of the case study, we randomly selected one of the two health centers and two associated health posts in each of the six project districts for a total of 6 health posts and 12 health centers [6].

### Study team

The study team was comprised of a U.S. based senior researcher and study principal investigator, an Ethiopian senior researcher, both of whom are medical anthropologists. The Ethiopian senior research supervised two field teams comprised of trained qualitative researchers, one for each of the study regions. Each field team had one male and one female member.

### Sampling and recruitment

The sample was comprised of adult women who perceived and survived excessive bleeding during or after birth and the witnesses to the event (e.g., the woman’s mother, her mother-in-law, her husband, other relative or neighbor. This group was designated as a “case.” In addition, we identified up to 6 women who died within 42 days of birth. We used purposive sampling and aimed for 24 cases, evenly distributed across the six districts: three mothers who perceived excessive bleeding after birth and one mother who died from any maternal complication in each of the six study districts. The inclusion criteria were: women aged 18–49 years, gave birth within the previous 6 months, resided in the MaNHEP project area, perceived and possibly experienced excessive bleeding or, in the case of maternal death, any complication, and willing and able to participate.

The field research teams identified potential cases from birth and postnatal registers at the sampled health centers and health posts, and by consulting Health extension workers, leaders of the Health development army and community members. The teams faced challenges in identifying the desired number of cases due to incomplete health records, lack of vital statistics, cases that were never brought to health facilities, and possible reluctance to report cases of illness and deaths.

Health development army and local community administration members assisted the field research teams by informing and scheduling appointments with potential respondents. The field research teams then met and screened each potentially eligible respondent (or family in the case of a maternal death) to determine if the mother met the inclusion criteria. To screen for perceived as well as possible experience of excessive bleeding, the women were asked if they had experienced any of the following symptoms after birth: continuous bleeding of any amount, passage of large or fist-sized blood clots, extreme weakness and/or fainting, or the care provider diagnosed excessive bleeding. The MaNHEP project’s community maternal and newborn health education intervention taught that these symptoms were danger signs for excessive bleeding that should trigger care seeking [8, 9].

The final sample consisted of 22 mothers -- 17 who perceived and possibly experienced excessive bleeding after birth and five who became ill and died from other causes. Given the aim of the study, we did not obtain medical diagnoses in the cases of perceived excessive bleeding or death from other causes.

The research teams invited mothers who met the inclusion criteria to participate in the study and then obtained informed consent using standard disclosure procedures.

### Data collection

An illness narrative is a qualitative rendering of an illness event by individual who experienced or witnessed the illness [10, 11]. Illness narratives by the .affected women, family caregivers and witnesses were used to collect data for the study. In cases of maternal death, caretakers and witnesses served as interviewees. Senior researchers and principal investigators from the five TRAction project country teams developed an illness narrative interview guide as part of the common research protocol. The guide, informed by the Integrated Illness History [10], McGill Illness Narrative Interview [11], and the Delay Model [7] was comprised of a standard pattern of open- and closed-ended questions and a systematic method of recall. It began with a request to provide a general narration of the illness event, followed by a series of questions to fill out the narrative with pertinent aspects of illness recognition and care seeking. These questions focused on perceptions of symptoms, severity and causes of symptoms, type and sequence of care received, decisions and considerations around care seeking, barriers and facilitators to and timing of care seeking steps, and finally the resolution of illness. The guide also contained a timeline to record and clarify the sequence of events, stimulate recall, and verify the illness narrative.

The field research teams elicited the narratives, one team in each of the two regions. They conducted the interviews in the homes of the woman affected by the illness, where the woman, a primary caretaker, witnesses and the interviewers were present. While the affected woman or a primary caretaker served as the main interviewee, other witnesses were able to add to or express their differences with the opinions of the former. One team conducted the interviews in Amharic in Amhara region, whereas the other team conducted the interviews using a female Oromiffa/Amharic translator in Oromia region. In each case, one team member elicited the narrative while the other one took field notes and completed the time line. In addition the teams used audiotape recorders to document the interviews. The narratives lasted about 1 to 1.5 h on average.

Within 4 h after an illness narrative interview, the teams held a debriefing session in which they used a template to document preliminary findings, their impressions of the interview, and the quality of the data obtained. Subsequently, they spent a day developing expanded field notes from memory, the debriefing field notes and the audiotape recordings. Expanded field notes capture details of the interview in the voice of the respondent and include verbatim quotations and relevant local language terms with direct translations. It captures interviewer and note taker comments and observations regarding the informant and interview situation, which helps to contextualize responses. Expanded notes were translated into English from the audio-recordings of the interviews. Available time and resources did not allow for back translation. The teams developed the debriefing reports and draft expanded field notes in Microsoft Office ® Word and sent these to the Ethiopian senior researcher by e-mail. The senior researcher, in turn, provided feedback to the teams by e-mail and they used this feedback to produce final versions of the expanded field notes to be used in the analyses.

### Data quality

Data quality was enhanced through rigorous training of experienced qualitative interviewers by the Ethiopian senior researcher on the study objectives and on the illness narrative guide through repeated role-play and feedback; informed consent procedures; cross-checking consistency of responses received from multiple witnesses to the event (see above) recorded during the interview; use of a timeline for clarification, recall and verification; debriefing sessions on illness narrative dynamics and data quality, and regular feedback by the senior researcher on each case’s expanded field notes.

### Analysis

The five country TRAction senior researchers and principal investigators developed a codebook a priori based on the illness narrative guide content. It included code definitions and inclusion and exclusion criteria, and the main thematic areas based on the Delay Model described above [7]. The Ethiopian senior researcher pretested and revised the codebook and, on the basis of the revised codebook, developed a coding template in QSR International Pty. Ltd. © Nvivo10 qualitative data management software. The Ethiopian senior researcher trained one of the four field research team members on the use of the codebook and the template to code the data from the expanded notes. The senior researcher and field team member then independently coded two expanded field note reports, compared their results, and resolved any discrepancies in coding. Subsequently, the field team member coded the expanded field notes for each case, supplementing the codebook and template with new codes as needed during coding. The field team member regularly submitted the results to the senior researcher for review and feedback.

The Ethiopian senior researcher and U.S. senior research conducted the subsequent analysis. This entailed three steps including: (1) thematic content analysis in Nvivo10, (2) entering social and demographic characteristics and thematic codes into Microsoft Office ® Excel (e.g., for each of the 22 cases, the specific signs, causes, decision considerations, care seeking steps that were described by the respondents and (3) conducting simple descriptive analysis of respondents’ social and demographic characteristics and thematic code frequencies. The thematic content analysis identified and portrayed themes related to illness recognition and care seeking mentioned above. Subsequently, the quantitative data from the excel file was used to determine the frequency of perceived symptoms, severity, causes, main decision makers, decision considerations, care seeking steps taken across cases. For example, if in an individual case, the symptom “continuous bleeding” was mentioned at least once, it was noted with a frequency of 1, even though this symptom may have been mentioned during the narrative and subsequently coded multiple times. If the symptom “heavy bleeding” was not mentioned, it was noted with a frequency of 0. The presentation of qualitative and quantitative data in the Results section below is aimed at providing both insight into and a sense of the prevalence of aspects of illness recognition and care seeking behavior.

## Results

### Sample characteristics

Half of the mothers were between 19 and 29 years of age (50%),about one-third of the mothers were between 30 and 39 years of age (32%), whereas 18% were more than 40 years of age. The majority of mothers (55%) had never attended school, with a higher proportion of these mothers residing in Oromia Region compared with their Amhara Region counterparts (64% versus 45%, respectively). Information on the literacy status of those who had not attended school was not collected. Some mothers (27%), however, had received more than 8 years of schooling. Most of the 17 mothers who survived gave birth at home (65%), others gave birth in a health center (29%), while one gave birth on the way to a health facility. Of the mothers who died, four died before birth and one died after birth. All mothers were in a health facility at the time of death.

### Illness recognition

#### Perceived symptoms and their severity

In order of frequency, many mothers and witnesses mentioned heavy or continuous bleeding (77%), fever (43%), unconsciousness (36%), severe headache and blood clots (32% each), drowsiness or dizziness (27%), and a cold body (23%). Between 10% and 20% of mothers and witnesses mentioned delayed placenta, stomach pain with bloating or heaviness, vomiting (18% each) and weakness (14%). Less than 10% of mothers and witnesses mentioned prolonged labor, weakness, dark blood, leg swelling and leg cramping, difficulty breathing and cough, backache, anemia, and baby not moving.

Indeed, heavy or continuous bleed was mentioned by 77% of women. From a biomedical perspective, other symptoms mentioned by at least 20% of mothers or witnesses were, with the possible exception of severe headache and fever, consistent with excessive bleeding. Other symptoms that may be associated with excessive bleeding are prolonged labor and delayed placenta as well as anemia. While mothers and witnesses may expect some bleeding to occur during or after a delivery, they recognized that it was abnormal by how heavy the bleeding was and by the occurrence of large blood clots or dark colored blood.

“The flow of blood was so much that the place and the mattress she was lying on were soaked with blood and it even sprayed on to us.” (Mother of a surviving mother, Amhara Region]

They also viewed as abnormal any bleeding that occurred before labor or birth; bleeding that continued beyond 3 days after birth; or excessive bleeding that occurred along with other symptoms such as loss of consciousness, headache, fever, chills, physical weakening, a delayed placenta or a pre-term birth.

Although the large majority of mothers and witnesses (68%) believed that the reported illness symptoms were serious, some were uncertain and changed their minds from not serious to serious (or vice versa) as the event unfolded (23%). Very few mothers and witnesses believed their symptoms were not serious (9%). Their assessments of illness severity were based on excess bleeding as in the quote above and other features of symptoms.

“It stopped on the fourth day so I was only a little worried, but I was thinking of going to the health facility if it [bleeding] continued like the first day because it may lead to my death.” [A mother who survived, Oromia Region]

“I was seriously worried about her. I thought she could die because when the bleeding increased, she became weaker and weaker.” [Father of a mother who survived, Oromia Region].

#### Perceived causes of symptoms

Mothers and/or witnesses mentioned a number of causes for the observed symptoms. In order of frequency, these were workload or heavy lifting (27%), evil spirits (23%), *mitch*, i.e., a condition to which a women is vulnerable if she goes outside and is exposed to the sun after engaging in preparation of food or within 40 days after birth (17%), and heredity or nature or a poor diet (14% each). Causes mentioned in less than 10% of cases include God’s punishment or fate, walking long distances, not drinking enough water or drinking coffee or local beer, poisoning, as well as pregnancy- and birth-related causes including pregnancy itself, births coming too close or too far apart, the birth of twins, a delayed placenta, bad birth practices, and absence of a birth assistant. Other causes mentioned by less than 10% of mothers and witnesses were cold weather, a lung problem and weakness.

The causes mentioned by mothers and/or witnesses might be categorized as “supernatural or metaphysical” in 32% of cases and “physical or biological” in 55% of cases. In some cases (14%), mothers and/or witnesses attributed the symptoms to both categories. Nearly half of mothers and/or witnesses reported multiple causes of the symptoms experienced (48%).

“I think it was because I didn’t have a good life. I worked really hard and I didn’t have a balanced diet which really hurt me over the long term.” (Surviving mother, Oromia Region)

“Teyazhua (an evil spirit) comes when you are angry. If a woman is angry, the spirit will come and kill her and drink her blood. It will kill her earlier than her predestined day.” [Mother of a surviving mother, Amhara Region].

“In our culture, there are evil people who call evil spirits. I suspect that someone did this to my daughter. How can she not feel better when she was taking that much medicine? Surprisingly, her blood pressure was normal, how can she die from a bad headache?” [Father of a mother that died, Amhara Region]

#### Perceived causes of death by family caregivers

Of the mothers that died, four died during pregnancy, undelivered. One was a mother who had experienced coughing and difficulty breathing. She was first referred to a health center and then on to a hospital. The witnesses had not initially viewed her symptoms as serious. They attributed her death to a “lung problem.” Another mother who had experienced leg cramping, feeling cold, and a lack of fetal movement died in a hospital. The witnesses had thought her symptoms were serious and attributed her death to pregnancy itself. The third mother that had experienced a severe headache, fever, vomiting, and swollen legs died in the hospital. The witnesses initially did not think her symptoms were serious but changed their minds. They attributed the mother’s death to “evil spirits.” A fourth mother had also experienced severe headache, fever and vomiting. She gave birth in a health center, was referred multiple times including to a hospital where she subsequently died. The witness did not initially think her symptoms were serious. They, too, attributed her death to “evil spirits.” Finally, the fifth mother who also had experienced a severe headache, fever, vomiting, along with stomach pain and bloating died in the hospital. Her symptoms were considered serious right away. Her death was thought to be caused by *mitch,* as well as heavy lifting.

### Care seeking

#### Decision-makers and time to decision to seek care

Those who made decisions about whether or not to seek care typically included the mother’s immediate family and close relatives. In Oromia Region, the mother herself, as well as neighbors and TBAs were also often involved. Among all who sought appropriate care, the estimated time from illness recognition to the decision to seek care ranged from immediately to within 1 h in all but two cases. In the two cases, the families waited 2 and 10 days, respectively, to decide to seek care.

#### Care seeking steps

Mothers and families pursued different pathways to receive care (Table 1). Of 17 mothers that survived the illness event, nine mothers sought biomedical care (53%). Of these nine mothers, all but one took between one and two steps to receive care. The one mother took a total of five steps to finally receive care. Eight mothers, however, did not seek biomedical care. All received home care. Three of these mothers also called in a local lay or traditional care provider for care, one of whom continued with home care.

**Table 1:** Biomedical and non-biomedical care-seeking steps taken by 17 surviving mothers

1st Step	2nd Step	3rd Step	4th Step	5th Step
Biomedical				
Care				
Hospital (n = 1)				
Health Center (*n* = 3)	Home (*n* = 2)			
Home Call-In (n = 1)	Health Center (*n* = 1)			
Home (n = 4)	Health Center (*n* = 4)	Home (n = 1)	Private Clinic (n = 1)	Home (n = 1)
Non-Biomedical				
Care				
Home (*n* = 8)	Home Call-In (n = 3)	Home (n = 1)		

Of the five mothers that died, all sought biomedical care (Table 2). Two mothers went directly to a hospital, one died in the hospital while the other went home and then back to a private clinic where she died. The remaining three mothers took anywhere from three to six care steps, mostly shuttling between home, health centers and hospitals.

**Table 2:** Biomedical care-seeking steps taken by mothers and families of 5 mothers who died

1st Step	2nd Step	3rd Step	4th Step	5th Step	6th Step
Biomedical					
Care					
Hospital (n = 2)	Home (n = 1)	Private Clinic (n = 1)			
Health Center (n = 3)	Home (n = 2)	Health Center (n = 2)	Hospital (n = 1)		
			Health Center (n = 1)	Health Center (n = 1)	Hospital (n = 1)

#### Factors influencing decision-making and care seeking

The mothers and witnesses mentioned a number of factors that enabled, delayed or prevented their use of biomedical care. The most frequently mentioned enabler of appropriate care seeking was the counseling provided by health extension workers and other care providers (e.g, midwives and nurses). This usually took place during antenatal care visits. Others also encouraged mothers who experienced a complication to seek care (e.g., family as well as health development army members and traditional birth attendants). Proximity of a health center and fear of being accused for not using medical care were also mentioned as “facilitating” care seeking. In one case, a trained traditional birth attendant dispensed misoprostol and effectively resolved the problem so that additional care was not needed.

Factors that led to delays in appropriate care seeking included logistical issues such as bad weather, roads, and lack of transport, a poor cellular network or a lack of response to calls for an ambulance. Lastly, factors that prevented care seeking included the mother’s physical weakness after birth, restrictions on the mother’s movement associated with the *hamo* tradition of postpartum seclusion, fear of *mitch,* or lack of awareness of the need to seek care. Other barriers included logistical constraints such as difficulties faced in transporting women from their homes to ambulances due to lack of labor or money, difficult terrain, and the discomfort associated with local means of transport or darkness.

### Challenges in receiving care

The story, below, is a paraphrase of the husband’s narrative account of the challenges in accessing and receiving timely quality care, leading to his wife’s death.

“It was around the end of the 5th month of her pregnancy when she felt sick. On the day before she fell sick, there were a lot of people who were working on our farm so she was serving food and water to them. She was very well. She woke me up around 11 pm and told me that she felt a severe headache and something pulling downward in her stomach. I tried calling for the ambulance immediately but the network was bad so I called my friend who had a car but it was at the garage for service. I found another car through him at 12 pm and we arrived at the hospital around 12:15 pm.”

“They took her inside on a stretcher immediately after we arrived and gave her glucose and an injection right away. One of the nurses was a good person and treated her well. They were visiting her every hour. She was getting better around 2 am but she started having a fever, sweating and her leg became cold around 4:00 am. I told the health workers and they suggested an ultrasound scan of the fetus but I was not able to take her to the ultrasound room since I was alone. I asked them to help me but they were reluctant to do so. She also became thirsty but I was not able to buy water since it was late at night. I even went to the kitchen to ask for water but they said there was no clean water. I was able to take her to the ultrasound room at 6:30 am. After the scan however, her stomach became bloated and she began to grunt.”

“The doctors arrived at the hospital around 8 AM even though they are assigned to work at night. One of them examined her at 8:30 AM and determined that the fetus had died. He decided to have her deliver the dead fetus vaginally but she was not able to deliver until 10 AM and became weaker and weaker. He finally decided to remove the fetus surgically. I went to another room to give blood but the health worker received a call from the surgeon and stopped taking blood from me. I returned to her room where I found her shouting loudly. She died soon after at 11:30 AM. They waited too long… .”

## Discussion

Main findings of this study are summarized below:

Most of the surviving mothers who perceived excessive bleeding recognized the meaning and severity of their symptoms.

A majority (53%) of these mothers (and their caregivers) sought timely biomedical care. Moreover, while three of five families of mothers who died failed to initially recognize the mother’s symptoms as serious (e.g., severe headache, vomiting), *all* sought timely biomedical care once they recognized that the mother’s condition was serious. Although the samples are not comparable, that 64% of all the mothers and families sought biomedical care in this study, compared with 45% of mothers who sought biomedical care for perceived major maternal complications observed in the 2012 MaNHEP end line survey [5] is encouraging.

For a variety of reasons, including inability of the health care staff at facilities to provide adequate care, many mothers (and families of the mothers who died) took multiple steps for the mother to finally receive care. This led to delays in care and, in the case of several mothers who died, may have contributed to their deaths. Surprisingly, perceived poor quality of care at health facilities did not appear to have been a barrier to care seeking, a finding that differs from what others have observed in terms of disrespectful maternity care [12–15].

Other studies have shown that knowledge or recognition of maternal danger signs is often low or variable [16–23] and that responses to maternal illness frequently involves multiple care steps, including the use of various home remedies, self-treatment with drugs (e.g., misoprostol) and formal biomedical care [20, 23]. As described above, however, in this study, recognition of excessive bleeding was found to be quite good (though this is not true for those symptoms resulting in the maternal deaths) although initial assessments of symptom severity varied.

Factors that facilitated care seeking included counseling by health workers and other community volunteers as well as proximity to health services. Counseling, whether in the context of antenatal care or community-based health education activities, and proximity to health facilities have both been documented by others [24–26]. Similarly, factors that impeded care seeking in this study, such as geographical, financial logistical barriers and cultural norms and practices have been well documented [19–30].

The strengths of this study include the use of the illness narrative method to generate data on illness recognition and care seeking that is grounded in actual experience and multiple perspectives of those involved in the event. This has enhanced the validity of the data. The collection of data on people’s perceptions, actions, the nature and context of decision-making, and social interactions has enabled us to gain a holistic sense of the factors that enable or constrain care seeking. The use of an illness event timeline and iterative neutral probes when eliciting the narrative stimulate recall and increase the validity of the data i.e., with respect to a collective memory of the event. Whether the method would elicit the same narrative if repeated is unknown. The narrative method requires adequate interviewer training in elicitation techniques and support as the narratives often evoke painful memories. It is possible that the involvement of two male fieldworkers in collecting the data may have influenced the type and extent of data received on birth complications especially from female respondents. However, the extensive experience of these fieldworkers on health-related issues and the pre-fieldwork discussions on sensitivity and creating rapport might have mitigated the effects of this potential constraint. The quality of the data enhanced our belief in the likelihood that this was the case.

The sampling procedures involving health the use of facility registers and consultations with community members have enabled the capture of most cases of severe maternal bleeding and death. On the other hand, the study’s 9-month time frame placed constraints on the study’s focus, method of case identification and on sample inclusion criteria. For example, participating senior researchers and principal investigators selected postpartum hemorrhage as a focus of the illness narratives of surviving mothers because it is one of the most common causes of maternal mortality in each setting and to simplify comparison of findings across the five countries. Near-miss case identification in health facilities would have been ideal but difficult to achieve given anticipated low referral rates. The group also elected to exclude young women less than 18 years of age because of concerns about issues surrounding consent and assent in low resource settings and the need for timely ethical review and approval at multiple levels—international, national and regional. As a result, the findings largely provide insights on recognition of and care seeking for excessive bleeding, as well as for any cause in the cases of maternal death, among adult women 18 years and older. We believe that this information has important implications for improving care seeking and care provision in the Ethiopian context.

## Conclusions

Mothers and family caregivers are able to recognize and seek timely biomedical care for abnormal bleeding, and also for less obvious symptoms of illness. These achievements can be reinforced through continued and focused health education and counseling, reduction of known barriers to care seeking, and improvements in the capacity of the health system to respond to maternal complications with high quality basic and comprehensive emergency obstetric care.

## Abbreviations

MaNHEP: Maternal and Newborn Health in Ethiopia Partnership
URC: University Research Company, LLC
USAID: United States Agency for International Development
